# Cytodiagnosis of multiple myeloma presenting as orbital involvement: a case report

**DOI:** 10.1186/1742-6413-3-19

**Published:** 2006-08-10

**Authors:** Alok Sharma, Manju Kaushal, Nishith K Chaturvedi, Rajbala Yadav

**Affiliations:** 1 Department of Pathology, Dr. Ram Manohar Lohia Hospital, New Delhi, India

## Abstract

**Background:**

Plasma cell neoplasms represent autonomous proliferations of plasma cells and can manifest as diffuse myeloma with systemic involvement (plasma cell myeloma or multiple myeloma), monoclonal gammopathy of undetermined significance (MGUS), or as variants of plasma cell myeloma such as indolent myeloma, smoldering myeloma, osteosclerotic myeloma, plasma cell leukaemia and non-secretory myeloma. Localized neoplastic proliferation of plasma cells presents as solitary plasmacytoma of bone or extramedullary plasmacytoma. Involvement of orbit can occur as a solitary plasmacytoma, or as part of systemic involvement in multiple myeloma, the clinical outcome being significantly worse in the latter setting.

Orbital involvement in multiple myeloma is very rare with less than 50 cases reported in the literature. Early cytological diagnosis of such lesions is vital for timely institution of appropriate therapy. As far as we are aware only six previous cases of cytological diagnosis of multiple myeloma involving the orbit are on record.

**Case presentation:**

A 37 year old male presented with low grade fever showing evening rise, headache, diplopia and swelling in the right periorbital and temporal region. Imaging studies revealed destructive lesion of sphenoid, frontal bone and zygomatic arch with soft tissue component extending to infratemporal fossa and orbit. A fine needle aspirate from the temporal region swelling showed features of a plasmacytoma, and subsequent workup confirmed the presence of systemic disease. A final diagnosis of multiple myeloma with orbital involvement at presentation was made.

**Conclusion:**

Present case describes the extremely rare presentation of multiple myeloma with orbital involvement and highlights the utility of cytology in such lesions. Fine needle aspiration diagnosis of plasmacytoma at extramedullary sites offers an opportunity for non-invasive verification of systemic involvement, and thus plays a major role in early diagnosis and management of these patients.

## Background

Plasma cell neoplasms represent autonomous proliferations of plasma cells and can manifest as diffuse myeloma with systemic involvement (plasma cell myeloma or multiple myeloma), monoclonal gammopathy of undetermined significance (MGUS), or as variants of plasma cell myeloma such as indolent myeloma, smoldering myeloma, osteosclerotic myeloma, plasma cell leukaemia and non-secretory myeloma. Localized neoplastic proliferation of plasma cells presents as solitary plasmacytoma of bone or extramedullary plasmacytoma.

Multiple myeloma is a tumour of malignant plasma cells that secrete monoclonal paraprotein. It accounts for about 10% of hematological malignancies [1], with an incidence of 5.5 cases per 100,000 population. The median age at diagnosis is 70 years and only 3.4% of cases are diagnosed between 35–44 years of age [2]. Orbital involvement as a manifestation of multiple myeloma is very rare. Less than 50 cases of such an involvement are described in the literature [3-7].

In recent years, fine needle aspiration cytology (FNAC) has emerged as the first line investigation in the evaluation of clinically palpable masses. Cytological evaluation of such lesions offers rapid and reliable diagnosis on which prompt treatment decisions can be made. As far as we are aware, only six previous cases of cytological diagnosis of orbital multiple myeloma are described in the literature [8-10].

We present the case of a 37 year old male presenting with low grade fever, proptosis, pain and redness in the right eye, and a diffuse swelling in periorbital and temporal region. FNAC from the temporal region swelling showed features of plasmacytoma and further investigations revealed systemic involvement by the disease (multiple myeloma).

## Case presentation

### Clinical history & examination

A 37 year old male presented with complaints of low grade fever with evening rise, malaise, anorexia and weight loss for 3 months, pain and redness in the right eye for 2 months, and diplopia and swelling in the right eyeball for 1 1/2 months.

General and systemic examinations were within normal limits. Ophthalmologic examination revealed a right proptosis and conjunctival chemosis. Visual acuity was normal. A diffuse tender swelling was present in the right periorbital and temporal region.

### Investigations

Routine hematological investigations showed mild normocytic normochromic anemia (Hemoglobin-9.7 g %) and an elevated Erythrocyte Sedimentation Rate (ESR) (124 mm/1^st ^hour). Other parameters were within normal limits.

Routine biochemical investigations did not reveal any abnormality.

**Figure 1:**
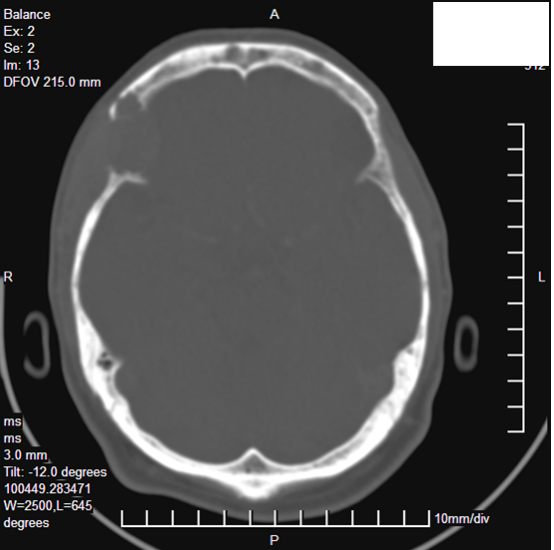
CECT scan showing destruction of right sphenoid, frontal bones and zygomatic arch with associated soft tissue component extending laterally into the infratemporal fossa and medially into the orbit.

**Figure 2:**
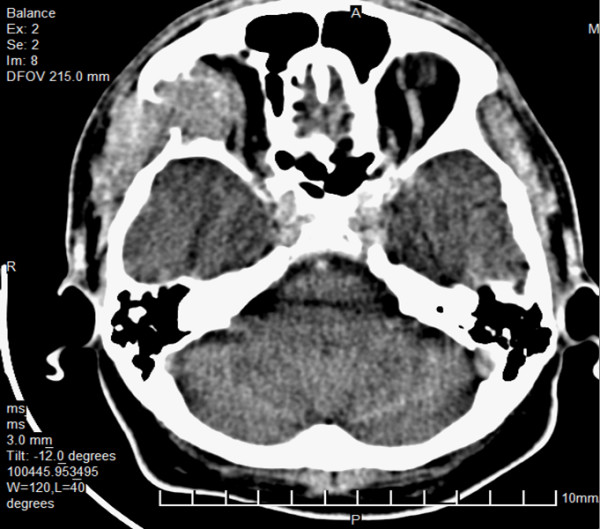
CECT scan showing destruction of right sphenoid, frontal bones and zygomatic arch with associated soft tissue component extending laterally into the infratemporal fossa and medially into the orbit.

**Figure 3:**
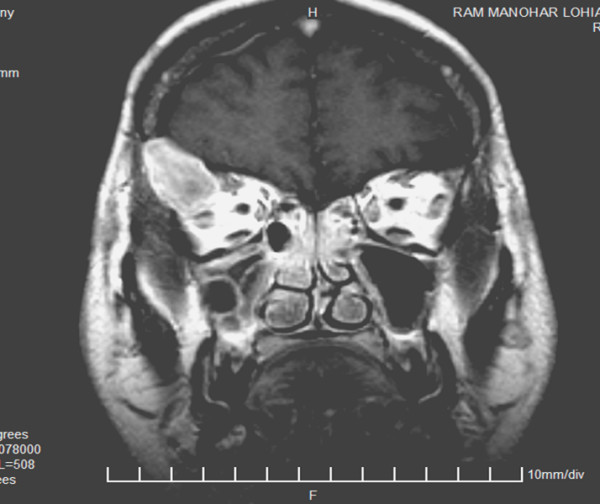
MRI scan showing destruction of right sphenoid, frontal bones and zygomatic arch with associated soft tissue component extending laterally into the infratemporal fossa and medially into the orbit.

Contrast Enhanced Computerized Tomography (CECT) [Figure 1 and 2] and Magnetic Resonance Imaging (MRI) [Figure 3] of head showed a lytic destructive lesion involving the right sphenoid bone, frontal bone and zygomatic arch. An associated soft tissue component was seen extending laterally into the infratemporal fossa and medially into the orbit.

Based on the clinico-radiological findings, diagnostic possibilities of a tubercular lesion, lymphoma and a sphenoid wing meningioma were considered.

FNAC from the diffuse swelling in the temporal region was performed using a 23 gauge needle attached to a 20 cc syringe. The aspirate was hemorrhagic. Smears fixed in 90% alcohol and air dried smears were stained by Papanicoloau technique, and Giemsa stain respectively.

**Figure 4:**
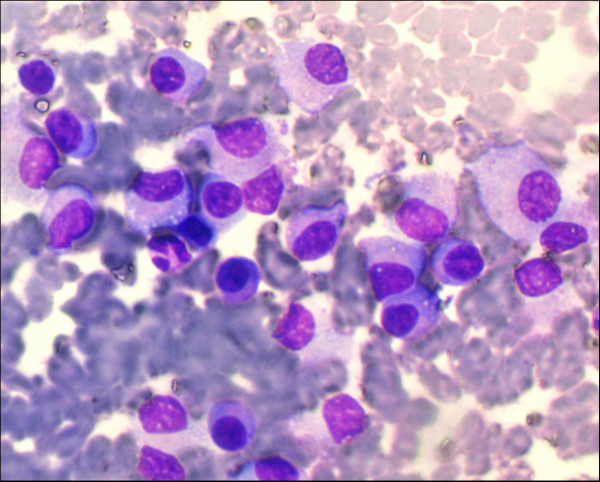
FNAC smears showing group of plasma cells showing eccentrically placed nucleus with cartwheel chromatin pattern and basophilic cytoplasm.(Giemsa × 100)

**Figure 5:**
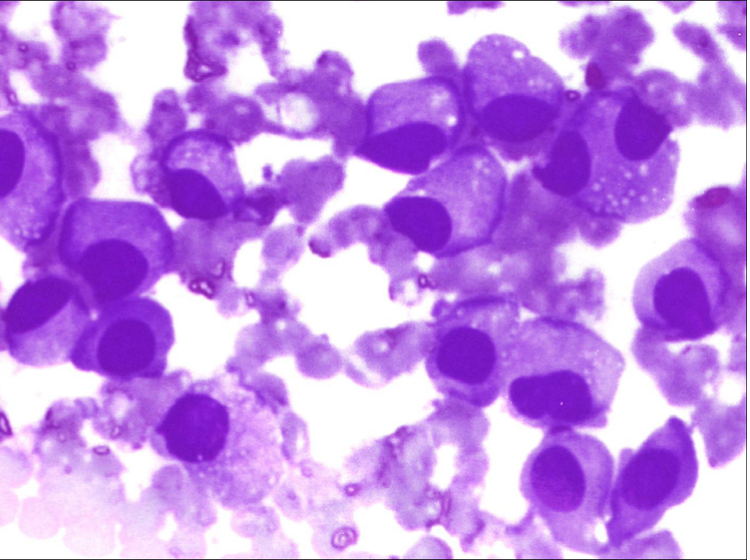
FNAC smears showing plasma cells with cytoplasmic vacuoles and binucleation in few cells. (Giemsa × 400).

Microscopic examination revealed hemorrhagic smears exhibiting single cells as well as groups of plasma cells having eccentrically placed nuclei with cartwheel chromatin pattern and basophilic cytoplasm with variable degree of paranuclear clearing [Figure 4]. Few cells also showed cytoplasmic vacuoles and binucleation [Figure 5]. A cytological diagnosis of plasmacytoma was thus given and further evaluation advised to rule out systemic involvement.

A detailed workup was performed. Test for urinary Bence-Jones proteins on a random urine sample was negative. Serum proteins showed a reversed albumin/globulin ratio (0.58). Serum calcium level (9.5 mg/dl) and renal function tests were normal. Serum beta-2 microglobulin was elevated (3788 μg/L; normal range 700–3400 μg/L).

Serum protein electrophoresis showed hypergammaglobulinemia and a monoclonal gammopathy (M spike) in the gamma globulin region (4.03 g/dL).

**Figure 6:**
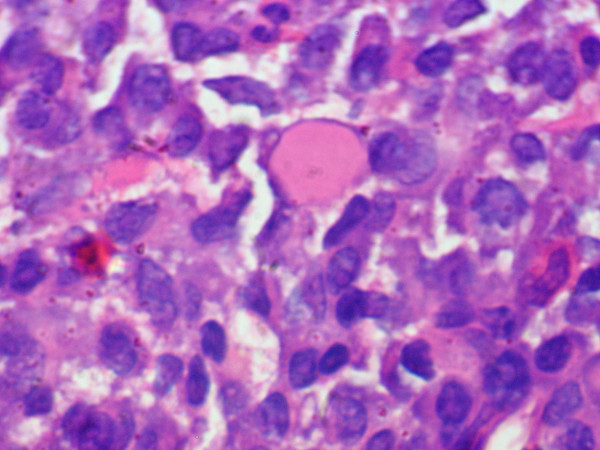
Photomicrograph of bone biopsy showing sheet of plasma cells with a prominent 'Russell body' (Hematoxylin & Eosin × 400).

Bone marrow aspiration and biopsy [Fig 6] showed infiltration by groups and sheets of plasma cells comprising about 40% of marrow population.

A skeletal survey did not show evidence of any other bony involvement.

A final diagnosis of multiple myeloma manifesting as orbital involvement at presentation was thus made.

## Discussion

Orbital involvement in multiple myeloma is a very rare finding. In four series of orbital tumors incidence of such a presentation varied from 1 in 200 to 1 in 800, and in 75% cases, it was the first manifestation of systemic disease [5]. In a review by Howling et al., 37 cases were described [3]. Since then few more cases have been reported [4-7].

In most of the cases onset of symptoms is insidious with slowly progressing proptosis accompanied by pain, diplopia and visual impairment. Restriction of motion, particularly during abduction has also been described [11]. Intracranial extension may lead to papilloedema and cranial nerve palsies [12]. Non-specific symptoms such as low grade fever, malaise and anorexia are common but can lead to an erroneous clinical impression in unsuspected cases, as in the present instance. Careful evaluation of the symptoms and performing the relevant investigations help in reaching an early diagnosis.

Imaging features of orbital multiple myeloma have been described in few cases. CT may show thinning of the overlying bone [13], or soft tissue masses arising within the bone causing marked bony expansion and destruction [6]. MRI usually shows low signal on T1 -weighted images and a high signal on T2-weighted images, but the findings may be variable [7].

Although cytological diagnosis of extramedullary plasmacytoma at various locations has been well described in several reports [14-16], a review of literature revealed only six previous cases of cytological diagnosis of an orbital involvement in multiple myeloma [8-10]. Since the clinical outcome is significantly worse in patients with systemic involvement as compared to those with solitary plasmacytoma, an early cytological diagnosis of extramedullary involvement in multiple myeloma helps in timely institution of appropriate treatment.

On microscopic examination, the finding of plasma cell proliferation indicates many diagnostic possibilities. A plasma cell myeloma may be apparent in cases where the plasma cells show significant cytologic atypia (i.e. polymorphous or blastic type). However in cases such as the present one, where plasma cells exhibit fairly mature cytologic features, possibilities of an extranodal marginal B-cell lymphoma with extensive plasmacytic differentiation and solitary plasmacytoma of bone need consideration. Further investigations such as flow cytometric analysis, protein electrophoresis/immunofixation, radiological studies and a bone marrow evaluation are required to confirm a diagnosis of plasma cell myeloma. This is particularly important as extramedullary plasmacytomas, which may represent marginal B-cell lymphomas with extensive plasmacytic differentiation have a significantly more favourable clinical course than solitary plasmacytoma of bone or plasma cell myeloma [17].

On fine needle aspirates, flow cytometry is often required to confirm the diagnosis of plasma cell myeloma and rule out the possibilities of extranodal marginal zone or lymphoplasmacytic lymphoma[18,19]. An international consensus group has recommended a panel of markers including cytoplasmic immunoglobulin light chains, CD38, CD45, CD56, CD19, CD20, immunoglobulin heavy chains and CD138 for multiparametric flow cytometric analysis in such cases [20]. Most of plasma cell myelomas are CD19-, CD45-/dim positive, CD38+++ and CD138+, while CD19 positivity is seen in lymphomas including the marginal zone lymphoma or lymphoplasmacytic lymphoma.

## Conclusion

Present case describes the extremely rare presentation of multiple myeloma with orbital involvement and highlights the utility of cytology in such lesions. Fine needle aspiration diagnosis of plasmacytoma at extramedullary sites offers an opportunity for non-invasive verification of systemic involvement and thus plays a major role in early diagnosis and management of these patients

## Competing interests

The author(s) declare that they have no competing interests.

## Authors' contributions

**AS **conceived the study, performed the FNAC, diagnosed the case, performed literature search and drafted, proof read and scrutinized the manuscript.

**MK **participated in the diagnostic workup, helped in drafting the manuscript and proof reading.

**NKC **participated in the diagnostic workup, helped in drafting the manuscript and scrutinized the manuscript.

**RBY **helped in drafting the manuscript and diagnostic workup.

All authors read and approved the final manuscript.
